# Limited effects of antibiotic prophylaxis in patients with Child–Pugh class A/B cirrhosis and upper gastrointestinal bleeding

**DOI:** 10.1371/journal.pone.0229101

**Published:** 2020-02-21

**Authors:** Te-Sheng Chang, Ying-Huang Tsai, Yi-Heng Lin, Chun-Hsien Chen, Chung-Kuang Lu, Wen-Shih Huang, Yao-Hsu Yang, Wei-Ming Chen, Yung-Yu Hsieh, Yu-Chih Wu, Shui-Yi Tung, Yen-Hua Huang

**Affiliations:** 1 Department of Gastroenterology and Hepatology, Chang Gung Memorial Hospital, Chiayi, Taiwan; 2 College of Medicine, Chang Gung University, Taoyuan, Taiwan; 3 Department of Biochemistry and Molecular Cell Biology, College of Medicine, Taipei Medical University, Taipei, Taiwan; 4 Department of Pulmonary and Critical Care Medicine, Chang Gung Memorial Hospital, Chiayi, Taiwan; 5 Research Center for Cell Therapy and Regeneration Medicine, Taipei Medical University, Taipei, Taiwan; 6 Division of Colon and Rectal Surgery, Department of Surgery, Chang Gung Memorial Hospital, Chiayi, Taiwan; 7 Department of Traditional Chinese Medicine, Chang Gung Memorial Hospital, Chiayi, Taiwan; 8 Health Information and Epidemiology Laboratory of Chang Gung Memorial Hospital, Chiayi, Taiwan; 9 Graduate Institute of Medical Sciences, College of Medicine, Taipei Medical University, Taipei, Taiwan; 10 Center for Reproductive Medicine, Taipei Medical University Hospital, Taipei, Taiwan; 11 Comprehensive Cancer Center of Taipei Medical University, Taipei, Taiwan; 12 International PhD Program for Cell Therapy and Regeneration Medicine, College of Medicine, Taipei Medical University, Taipei, Taiwan; Epicentre, CAMEROON

## Abstract

Current guidelines recommend antibiotic prophylaxis for all patients with various degrees of cirrhosis and upper gastrointestinal (UGI) bleeding. This study assessed the need for antibiotic prophylaxis in patients with low Child–Pugh scores. We retrospectively screened all patients with cirrhosis who underwent upper endoscopies for UGI bleeding in a referral hospital in Taiwan between 2003 and 2014, from which 913 patients were enrolled after excluding patients with active bacterial infections, recent antibiotic use, early death, and Child–Pugh class C cirrhosis. Among them, 73 (8%) received prophylactic antibiotics, and 45 (4.9%) exhibited 14-day bacterial infection. Neither Child–Pugh score nor model for end stage liver disease score were optimal for predicting bacterial infection because their areas under the curves were 0.610 (95% confidence interval [CI]: 0.529–0.691) and 0.666 (95% CI: 0.591–0.742), respectively. Antibiotic prophylaxis did not reduce the risks of 14-day bacterial infection (relative risk [RR]: 0.932, 95% CI: 0.300–2.891, P = 0.902), 14-day rebleeding (RR: 0.791, 95% CI: 0.287–2.181, P = 0.650), or 42-day mortality (RR: 2.710, 95% CI: 0.769–9.524, P = 0.121). The results remained similar after propensity score adjustment. On-demand antibiotic treatment might suffice for patients with Child–Pugh class A/B cirrhosis and UGI bleeding.

## Introduction

Patients with cirrhosis are considered immunocompromised and susceptible to various bacterial infections that may aggravate the major complications of cirrhosis [1]. Therefore, preventing infections is considered a constitutive part of patient care, and all the relevant practice guidelines and consensus statements recommend antibiotic prophylaxis for all patients with cirrhosis in specific settings, including secondary prophylaxis for patients with previous spontaneous bacterial peritonitis (SBP) and primary prophylaxis for patients with poor liver function and those with active upper gastrointestinal (UGI) bleeding [2–4]. The role of prophylactic antibiotics is critical in patients with cirrhosis and UGI bleeding because bacterial infections during or immediately after bleeding episodes are associated with severe complications, such as failure to control bleeding, rebleeding, and mortality during hospitalization [5–7].

Prophylactic antibiotics have substantial benefits in patients with cirrhosis and UGI bleeding; consequently, prophylactic antibiotics have been endorsed by major clinical practice guidelines [2–4]. However, excessive antibiotic use is associated with side effects, including drug toxicity, drug–drug interaction, and *Clostridium difficile* infection [8]. Moreover, a recent study demonstrated that antibiotic prophylaxis in hospitalized patients with cirrhosis may increase the risk of subsequent infection [9]. Therefore, a balance of the risks and benefits of routine and indistinctive prophylactic antibiotic use in all patients with cirrhosis and UGI bleeding is essential. In the 2000s, numerous patients with cirrhosis-related ascites did not receive recommended services, including prophylactic antibiotics, during UGI bleeding [10]. Taking advantage of this situation, the authors of the present study have observed that not all patients had identical bacterial infection risk without prophylactic antibiotic use.

In patients with cirrhosis and portal hypertension, risk stratification and personalized care have become primary strategies in the current stratified medicine era [4,11]. In recent years, patients with cirrhosis of varying severity may have disparate levels of risk of complications during UGI bleeding; therefore, such patients have varying needs for prophylactic antibiotics [12,13]. The risk of cirrhosis-related complications, including gastroesophageal variceal hemorrhage, mainly depends on the degree of portal hypertension, which is proportional to the severity of cirrhosis [14]. The Child–Pugh score is the simplest and most effective tool for assessment of bedside prognosis in patients with cirrhosis [12,15]. In this study, we reappraised whether a stratified, rather than indiscriminate, prophylactic antibiotic use strategy, based on the Child–Pugh score, is applicable in patients with cirrhosis and UGI bleeding.

## Materials and methods

### Study patients and data collection

In this retrospective study, we analyzed all patients with cirrhosis aged ≥ 20 years who underwent upper endoscopy for UGI bleeding between January 2003 and December 2014 at Chang Gung Memorial Hospital, a tertiary referral hospital in Chiayi, southern Taiwan. Data were retrieved from the hospital’s computer archives; the electronic medical records of all eligible patients were reviewed to obtain necessary data, including clinical data, demographic characteristics, and laboratory examination results. The study endpoints included 14-day infection and rebleeding rates and 42-day mortality rate. All patient records were traced until day 42 (end of week 6) or death. The exclusion criteria were (1) bacterial infection on the day of UGI bleeding, (2) antibiotic use within 1 week before UGI bleeding, (3) Child–Pugh class C (score ≥ 10), and (4) death within the first 24 h. To prevent the recording of redundant demographic variables, only the first UGI bleeding episode in patients with multiple UGI bleeding episodes was included for analysis as the index bleeding episode. Because the present study was retrospective without specific interventions for the study patients, the requirement for informed consent was waived. All patient data were fully anonymized before access and this study was approved by the Chang Gung Medical Foundation Institutional Review Board (IRB No: 201701786B0).

### Management program

Since the inauguration of Chang Gung Memorial Hospital, Chiayi branch (i.e., the study site) in 2002, upper endoscopy using a video endoscope (GIF-Q240Z, Olympus, Tokyo, Japan) and an electronic endoscopic system, either the EVIS 240 series (Olympus) or the EVIS 260 series (Olympus) with endoscopic hemostasis within 24 h (usually within 12 h) has been the standard of care for all patients with UGI bleeding due to any etiology. Endoscopic hemostasis is achieved using measures, including band ligation, injection sclerosis, and coagulation, depending on the clinical scenario and operators’ decisions. The vasoactive drug terlipressin (Glypressin, Ferring AB, Malmö, Sweden) with or without intravenous proton pump inhibitor (PPI) use is generally initiated before endoscopy if the medical history and clinical picture suggests portal hypertension. After endoscopy, patients receive terlipressin at 1 mg/6 h for 3 days for UGI bleeding due to portal hypertension and intravenous PPI (omeprazole or pantoprazole 80 mg bolus loading and 40 mg twice daily for 1–3 days) due to peptic ulcers. Oral PPI is given for 2–4 months for patients with peptic ulcer disease. Patients may undergo repeat endoscopy or surgical devascularization if the initial endoscopic therapy fails. Secondary prophylaxis with the oral β-blocker propranolol is initiated as soon as variceal bleeding stops, provided no contraindication is noted. Decisions regarding the use and choice of prescribed antibiotics are taken by the clinicians in charge, most of whom are from the emergency department.

### Definition

We defined antibiotic prophylaxis as antibiotic prescription following bleeding in the absence of clinical signs of bacterial infection. Liver cirrhosis was diagnosed on the basis of the presence of esophageal or gastric varices observed through endoscopy with compatible image characteristics and clinical features indicating portal hypertension, such as splenomegaly, hepatic dysfunction, or thrombocytopenia. The patients were classified according to liver disease etiologies, namely hepatitis B virus (HBV) hepatitis (presence of the HBV surface antigen in the serum), hepatitis C virus (HCV) hepatitis (presence of the HCV antibody in the serum), dual HBV and HCV hepatitis (BC) hepatitis (presence of both HBV and HCV), and non-BC hepatitis (NBNC; negative for both HBV and HCV). The cause of UGI bleeding, either portal hypertension (gastric or esophageal varices) or peptic ulcers, was determined after endoscopy revealed active bleeding or stigmata of recent hemorrhage accompanied by a reduction in hemoglobin levels. Rebleeding was diagnosed when bleeding recurred more than 24 h after the initial bleeding episode had been controlled. The diagnoses of bacterial infections were made based on the standard guidelines [16], In addition, SBP was defined as an ascites polymorphonuclear cell count of ≥250 cells/mm^3^ and/or positive ascitic fluid culture. Common skin flora, particularly coagulase-positive staphylococci, was considered contaminants unless blood cultures were positive on two or more occasions or clinical signs of the relevant infection were observed.

### Statistical analysis

Continuous variables are expressed as means ± standard deviations. The qualitative and quantitative differences between the groups were analyzed using the chi-square or Fisher exact test for categorical parameters and the Student *t* test for continuous parameters. The multivariate logistic regression model was used to identify independent risk factors. Receiver operating characteristic (ROC) curves were employed to assess the performance characteristics of the Child–Pugh and model for end-stage liver disease (MELD) scores in predicting the acquisition of infection. All statistical tests were performed using SPSS (version 22.0; IBM, Chicago, IL, USA). A two-tailed *P* < 0.05 was considered significant. Because antibiotics are theoretically more likely to be prescribed to patients with more severe cirrhosis, propensity scores (PPS) were calculated using antibiotic prophylaxis as the outcome variable in a multivariate logistic regression model.

## Results

### Patient characteristics

**Fig 1:**
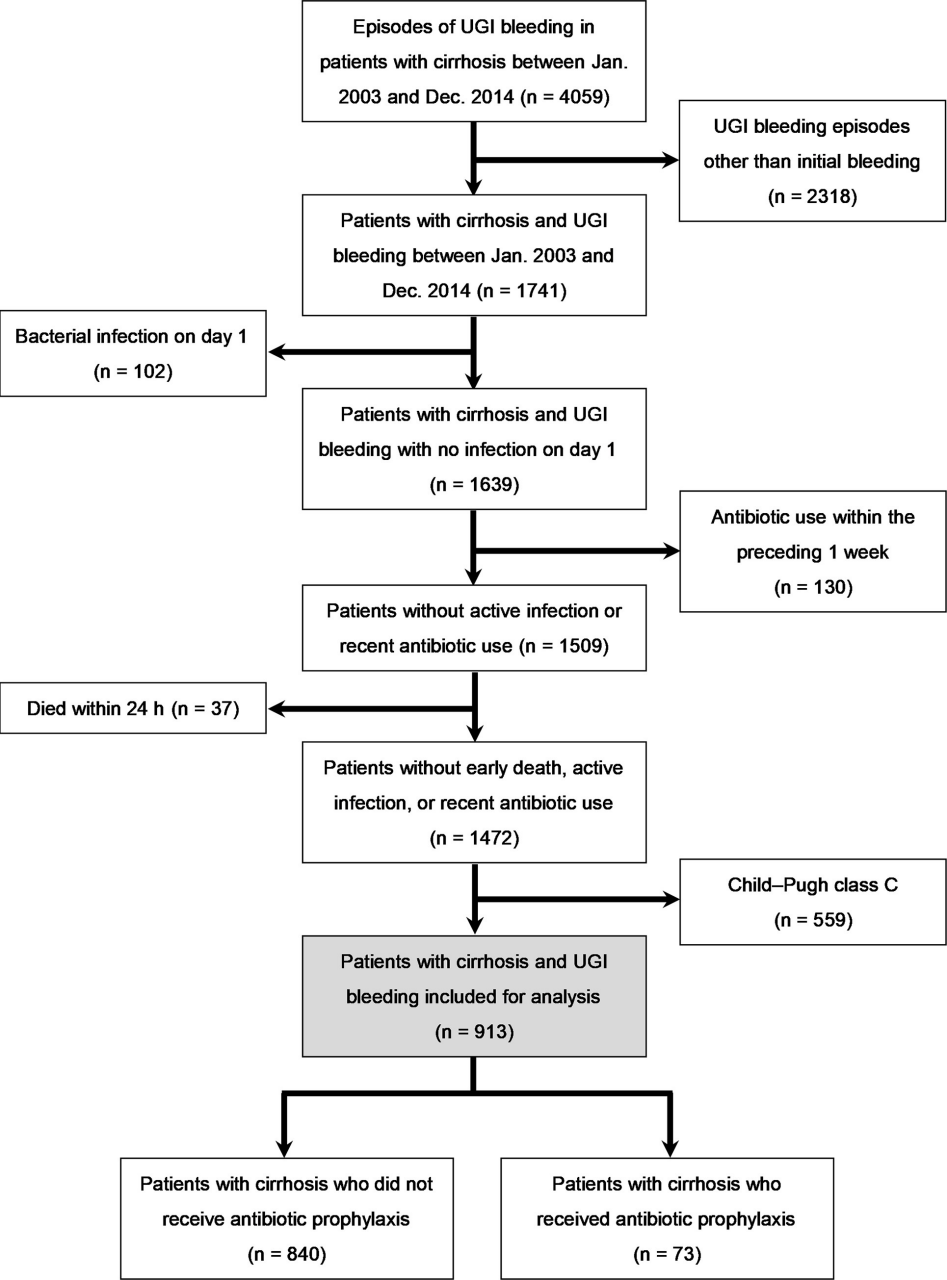
Flow of patient enrollment.

Fig 1 presents the flow of all included patients. In total, 1741 patients presented to the study site and had 4059 UGI bleeding episodes between January 2003 and December 2014. After the exclusion of 102 patients with active bacterial infections, 130 with recent antibiotic use, 37 who died within 24 h, and 559 with Child–Pugh class C scores, the remaining 913 were enrolled for analysis. Of these 913 patients, 73 received antibiotic prophylaxis and 840 did not. Of the 73 patients who received antibiotic prophylaxis, 19 received cefazolin, 4 received cefazolin plus gentamicin, 6 received cefuroxime, and 44 received ceftriaxone. The baseline characteristics of these 913 patients with Child–Pugh A/B scores are listed in Table 1. Their mean age was 59.49 ± 13.15 years, and the male-to-female ratio was 2.42:1. Those who received antibiotic prophylaxis were younger (55.22 ± 12.46 vs. 59.86 ± 13.15 years, *P* = 0.003), had more units of blood transfusion (3.38 ± 2.78 vs. 2.43 ± 2.44 units, *P* = 0.006), had higher white blood cell counts (9.87 ± 4.28 vs. 7.92 ± 4.18 ×10^3^/μL, *P* < 0.001), had lower systolic blood pressure (112.47 ± 27.24 vs. 121.37 ± 31.03 mmHg, *P* = 0.007), had a higher rate of intensive care unit admission (9.6% vs. 2.3%, *P* < 0.001), and had a higher rate of portal hypertension-related (variceal) bleeding (93.10% vs. 77.14%, *P* = 0.001). The patients who received and did not receive antibiotic prophylaxis did not differ significantly in terms of sex, presence of hepatocellular carcinomas (HCCs), Child–Pugh scores, MELD scores, prior SBP, duration of hospitalization, and rates of 14-day infection, 14-day rebleeding, and 42-day mortality. Because this study was conducted over a long 12-year period, we divided the patients into two groups according to the period of UGI bleeding to understand whether there were differences in patient characteristics over time. As shown in Table 2, compared with patients who presented between 2003 and 2008, patients who presented between 2009 and 2014 were older (58.43 ± 12.98 vs. 60.70 ± 13.26 years, *P* = 0.009), had a higher rate of prophylactic antibiotic use (2.9% vs. 13.8%, *P* < 0.001), had shorter duration of hospitalization (6.92 ± 5.24 vs. 6.21 ± 5.71 days, *P* = 0.05), and had a lower Child–Pugh score (7.40 ± 1.18 vs. 7.22 ± 1.22, *P* = 0.0027). A similar insignificant difference in rates of 14-day infection, 14-day rebleeding, and 42-day mortality was observed separately in both Child–Pugh class A and class B patients (S1 Table). We also analyzed the 716 patients with portal hypertensive bleeding as a separate group, and the baseline characteristics of these patients are listed in S2 Table.

**Table 1:** Baseline patient characteristics.

Patient characteristics		Prophylaxis	No prophylaxis	All patients	*P* ^†^
		(n = 73)	(n = 840)		
Age, mean ± SD (years)		55.22 ± 12.46	59.86 ± 13.15	59.49 ± 13.15	0.003
Sex, male *n* (%)		57 (78.1)	592 (70.5)	649 (71.1)	0.169
HCC, *n* (%)		19 (26.0)	287 (34.2)	306 (33.5)	0.158
Blood transfused in 48 h, unit		3.38 ± 2.78	2.43 ± 2.44	2.51 ± 2.48	0.006
Ascites, *n* (%)		33 (45.2)	344 (41.0)	377 (41.3)	0.479
Hepatic encephalopathy, *n* (%)		6 (8.2)	41 (4.9)	47 (5.1)	0.216
Prior SBP, *n* (%)		1 (1.4)	17 (2.0)	18 (2.0)	0.700
Etiology of cirrhosis, *n* (%)					0.211
	HBV	22 (30.1)	173 (20.5)	195 (21.3)	
	HCV	28 (38.4)	404 (48.2)	432 (47.2)	
	BC	8 (11.0)	77 (9.2)	85 (9.2)	
	NBNC	15 (20.5)	186 (22.1)	201 (22.3)	
Platelet count, ×10^3^/μL		114.51 ± 68.49	112.25 ± 70.31	112.43 ± 70.13	0.788
White blood cell count, ×10^3^/μL		9.87 ± 4.28	7.92 ± 4.18	8.06 ± 4.22	<0.001
Hemoglobin, g/L		9.37 ± 2.26	9.21 ± 2.52	9.22 ± 2.50	0.593
International normalized ratio		1.32 ± 0.17	1.27 ± 0.45	1.28 ± 0.44	0.351
Sodium, mEq/L		137.00 ± 4.00	136.56 ± 10.81	136.59 ± 10.42	0.724
Creatinine, mg/L		1.15 ± 0.76	1.28 ± 1.25	1.27 ± 1.22	0.379
Bilirubin, mg/dL		1.81 ± 1.04	1.78 ± 1.15	1.78 ± 1.14	0.829
ALT, IU/L		47.15 ± 24.89	65 ± 125.50	62.93 ± 120.40	0.229
Albumin, g/dL		2.97 ± 0.47	2.91 ± 0.55	2.91 ± 0.54	0.301
Systolic blood pressure, mmHg		112.47 ± 27.24	121.37 ± 31.03	120.62 ± 30.81	0.019
Heart rate, beats/min		105.01 ± 21.48	100.31 ± 21.60	94.68 ± 31.40	0.079
Etiology of bleeding, *n* (%)					0.001
	Portal hypertension	68 (93.1)	648 (77.14)	716 (78.4)	
	Peptic ulcer	5 (6.9)	192 (22.86)	197 (21.6)	
Hospitalization days		7.33 ± 6.79	6.50 ± 5.40	6.56 ± 5.53	0.218
ICU admission, *n* (%)		7 (9.6)	19 (2.3)	26 (2.84)	<0.001
MELD score		12.85 ± 3.84	12.53 ± 3.71	12.59 ± 3.79	0.480
Child–Pugh score		7.34 ± 1.20	7.32 ± 1.21	7.32 ± 1.21	0.855
Child–Pugh class A/B, *n* (%)		18/55	239/601	257/656	0.489
		(24.7/75.3)	(28.5/71.5)	(28.1/71.9)	
Infection within 14 days, *n* (%)		5 (6.8)	40 (4.8)	45 (4.9)	0.429
Rebleeding within 14 days, *n* (%)		5 (6.8)	71 (8.5)	76 (8.3)	0.634
Mortality within 42 days, *n* (%)		5 (6.8)	27 (3.2)	32 (3.5)	0.161

^†^ Comparison between antibiotic prophylaxis and no prophylaxis groups

*Abbreviations*: *SD*, standard deviation; *HCC*, hepatocellular carcinoma; *SBP*, spontaneous bacterial peritonitis; *HBV*, hepatitis B virus; *HCV*, hepatitis C virus; *BC*, presence of both HBV and HCV; *NBNC*, negative for both HBV and HCV; *ALT*, alanine aminotransferase; *ICU*, intensive care unit; *MELD*, model for end-stage liver disease.

**Table 2:** Changes in patient characteristics and results over time.

	2003–2008 (n = 487)	2009–2014 (n = 426)	*P* ^†^
Age, mean ± SD (years)	58.43 ± 12.98	60.70 ± 13.26	0.009
Sex, male *n* (%)	351 (72.1)	298 (70)	0.527
Prophylaxis, *n* (%)	14 (2.9)	59 (13.8)	<0.001
Hepatocellular carcinoma, *n* (%)	159 (32.6)	147 (34.5)	0.601
Blood transfused in 48 h, unit	2.45 ± 2.47	2.57 ± 2.48	0.475
Ascites, *n* (%)	203 (41.7)	174 (40.8)	0.850
Hepatic encephalopathy, *n* (%)	27 (5.5)	20 (4.7)	0.668
Platelet count, ×10^3^/μL	110.58 ± 76.93	114.55 ± 61.44	0.393
Hemoglobin, g/L	9.32 ± 2.31	9.10 ± 2.69	0.187
Creatinine, mg/L	1.28 ± 1.22	1.21 ± 1.21	0.762
Hospitalization days	6.92 ± 5.24	6.21 ± 5.71	0.05
MELD score	12.69 ± 3.76	12.40 ± 3.66	0.246
Child–Pugh score	7.40 ± 1.18	7.22 ± 1.22	0.027
Infection within 14 days, *n* (%)	33 (6.8)	12 (2.8)	0.09
Rebleeding within 14 days, *n* (%)	39 (8)	37 (8.7)	0.803
Mortality within 42 days, *n* (%)	18 (3.7)	14 (3.3)	0.722
Body mass index (kg/m^2^)	24.87 ± 3.71	24.85 ± 3.88	0.918
Diabetes mellitus, *n* (%)	163 (33.5)	169 (39.7)	0.061
ICU admission, *n* (%)	11 (2.3)	15 (3.5)	0.345

^†^ Comparison between patients enrolled during 2003–2008 and 2009–2014.

*Abbreviations*: *SD*, standard deviation; *ICU*, intensive care unit; *MELD*, model for end-stage liver disease.

### Factors associated with risk of 14-day infection

**Fig 2:**
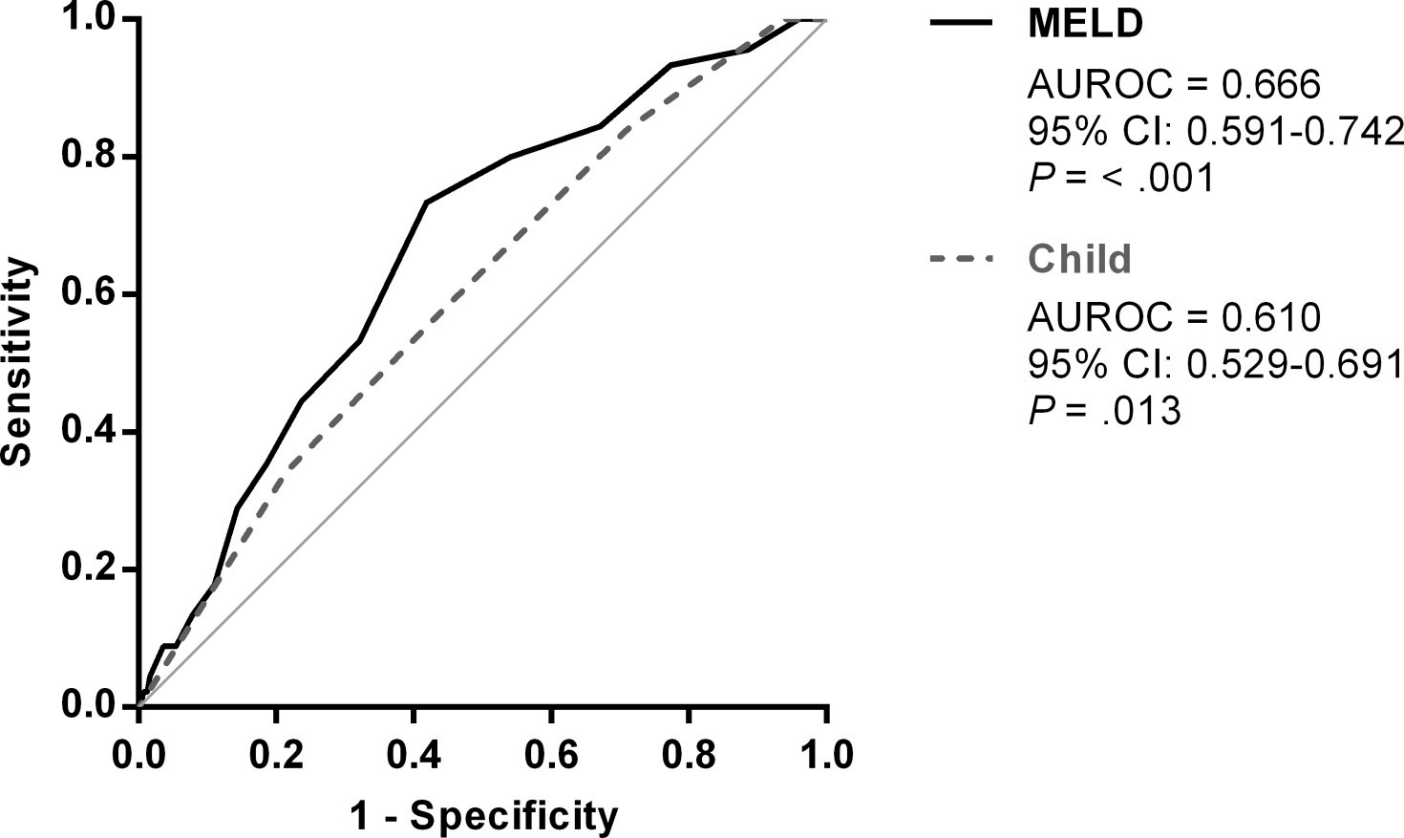
ROC curves for Child–Pugh scores of 5–9 and the corresponding MELD scores for predicting bacterial infection. The AUCs were 0.610 (95% CI: 0.529–0.691, *P* = 0.013) and 0.666 (95% CI: 0.591–0.742, *P* < 0.001) for the Child–Pugh and MELD scores, respectively. Abbreviations: ROC, receiver operating characteristic; AUC, area under the ROC curve; MELD, model for end-stage liver disease; CI, confidence interval.

During the 11-year observation period, only 45 (4.9%) of the enrolled 913 patients exhibited confirmed bacterial infection within 14 days of presentation at the hospital (Table 3). Table 4 lists bacterial infection types in these 45 patients. No significant reduction was observed in the risk of acquiring bacterial infection in the patients who received antibiotic prophylaxis compared with those who did not (relative risk [RR]: 0.932, 95% confidence interval [CI]: 0.300–2.891, *P* = 0.902). There was also no significant difference in infection risk with respect to prophylactic antibiotic use when Child–Pugh class A and class B patients were analyzed separately (S3 Table). Except for white blood cell counts (RR: 1.102, 95% CI: 1.016–1.194, *P* = 0.019), platelet counts (RR: 0.991, 95% CI: 0.983–0.998, *P* = 0.019), albumin levels (RR: 0.434, 95% CI: 0.195–0.965, *P* = 0.041), and MELD scores (RR: 1.132, 95% CI: 1.033–1.241, *P* = 0.008), all clinical and laboratory parameters—including age, sex, ascites, previous SBP, blood pressure, hemoglobin levels, presence of HCC, intensive care unit admission, Child–Pugh score, etiology of cirrhosis, cause of UGI bleeding, and endoscopic therapy—did not affect the RR of bacterial infection (Table 3). After PPS adjustment with antibiotic prophylaxis as the outcome variable, 138 patients who received and did not receive antibiotic prophylaxis were obtained at a ratio of 1:1, forming two groups with 69 patients each. Among the 138 patients, 9 had bacterial infection; risk of infection did not differ significantly between those who received and did not receive antibiotic prophylaxis (RR: 0.962, 95% CI: 0.147–6.288, *P* = 0.968). Fig 2 presents the ROC curves and areas under the ROC curves (AUCs) of the Child–Pugh and MELD scores for predicting bacterial infection. Among the patients with cirrhosis with Child–Pugh scores of 5–9 and UGI bleeding, neither the Child–Pugh score nor the MELD score was an optimal tool for predicting bacterial infection because the AUCs (95% CIs) of the Child–Pugh and MELD scores were 0.610 (0.529–0.691) and 0.666 (0.591–0.742), respectively—both <0.7. When only the 716 portal hypertensive bleeding patients were included, the risk of 14-day infection did not differ significantly between those who received and did not receive antibiotic prophylaxis (RR: 0.827, 95% CI: 0.233–2.937, *P* = 0.769) as shown in S4 Table.

**Table 3:** Factors associated with bacterial infection risk within 14 days^†^.

Factors		All patients (n = 913)			PPS-matched patients (n = 138)		
		RR	95% CI	*P*	RR	95% CI	*P*
Prophylaxis, y/n		0.932	0.300–2.891	0.902	0.962	0.147–6.288	0.968
Age, years		1.015	0.985–1.047	0.325	1.011	0.907–1.128	0.840
Sex, male/female		0.671	0.317–1.418	0.296	2.233	0.184–27.056	0.528
Prior SBP, y/n		1.213	0.138–10.682	0.862	–	–	0.999
Ascites, y/n		1.300	0.563–3.003	0.539	0.964	0.048–19.292	0.981
HCCs, y/n		1.197	0.592–2.419	0.616	0.394	0.032–4.869	0.468
Blood transfusion, unit		1.011	0.874–1.169	0.885	0.919	0.619–1.364	0.676
Encephalopathy, y/n		1.084	0.282–4.169	0.906	0.070	0.001–6.825	0.255
Blood pressure, mmHg		1.001	0.989–1.012	0.930	1.024	0.985–1.064	0.224
Hemoglobin, g/L		1.119	0.933–1.342	0.227	1.156	0.611–2.187	0.655
WBC count, ×10^3^/μL		1.102	1.016–1.194	0.019	1.260	0.832–1.910	0.275
Platelet count, ×10^3^/μL		0.991	0.983–0.998	0.019	0.955	0.908–1.005	0.078
Albumin, g/dL		0.434	0.195–0.965	0.041	0.920	0.055–15.374	0.954
ICU admission, y/n		0.298	0.077–1.145	0.078	17.131	0.557–526.696	0.104
MELD score		1.132	1.033–1.241	0.008	0.979	0.665–1.441	0.913
Child Pugh score		0.890	0.563–1.409	0.619	3.514	0.489–25.242	0.212
Etiology of cirrhosis				0.670			0.767
	NBNC	1.000			1.000		
	HBV	2.046	0.641–6.532	0.227	1.220	0.062–24.122	0.896
	HCV	1.693	0.573–5.001	0.341	3.477	0.242–49.912	0.359
	BC	1.395	0.301–6.463	0.670	–	–	0.998
Treatment				0.544			0.988
	No treatment	1.000			1.000		
	APC	1.892	0.496–7.209	0.350	1.150	0.008–170.674	0.956
	EVL	0.929	0.385–2.242	0.870	0.728	0.064–8.229	0.798
	EIS	1.395	0.301–6.463	0.670	1.058	0.082–13.609	0.965
Etiology of bleeding				0.250			0.999
	Portal hypertension	1.000			1.000		
	Peptic ulcer	0.468	0.128–1.705	0.250	–	–	

^†^ Number of patients with 14-day infection: 45 of all 913 patients and 9 of the 138 PPS-matched patients.

*Abbreviations*: *PPS*, propensity score; *RR*, relative risk; *CI*, confidence interval; *y/n*, yes/no; *SBP*, spontaneous bacterial peritonitis; *HCC*, hepatocellular carcinoma; *WBC*, white blood cell; *ICU*, intensive care unit; *MELD*, model for end-stage liver disease; *NBNC*, negative for both HBV and HCV; *HBV*, hepatitis B virus; *HCV*, hepatitis C virus; *BC*, presence of both HBV and HCV; *APC*, argon plasma coagulation; *EVL*, endoscopic variceal ligation; *EIS*, endoscopic injection sclerosis.

**Table 4:** Types of bacterial infection within 14 days (n = 45).

Type	No	Site and bacteria	
SBP	7	Blood	Ascites
	1	*Bacteroides thetaiotaomicron*	*Bacteroides thetaiotaomicron*
	2	*Escherichia coli*	–
	1	Group B streptococci	–
	1	–	Viridans streptococci
	2	–	–
Pneumonia	7	Blood	Sputum
	2	–	*Haemophilus influenza*
	1	–	*Acinetobacter baumannii*
			ORSA
	1	*Staphylococcus aureus*	–
	1	*Staphylococcus aureus*	–
		Viridans streptococci	
	2	–	–
UTI	12	Blood	Urine
	1	*Acinetobacter baumannii*	–
		*Escherichia coli*	
	1	–	Gram-positive cocci (2 types)
	1	*Klebsiella pneumoniae*	*Klebsiella pneumoniae*
	1	*Acinetobacter baumannii*	*Serratia marcescens*
	1	–	Coagulase-negative staphylococci
	1	–	*Klebsiella pneumoniae*
	1	*Escherichia coli*	–
	1	*Bacillus* spp.	*Enterococcus faecalis*
	1	*Escherichia coli*	*Escherichia coli*
	3	–	–
Cellulitis	2	Blood	
	1	*Staphylococcus aureus*	–
	1	*Klebsiella pneumoniae*	–
Arthritis	2	Blood	Synovial fluid
	1	–	*Staphylococcus aureus*
	1	Group B streptococci	–
Unknown	15	Blood	
	1	*Stenotrophomonas maltophilia*	–
	2	*Klebsiella pneumoniae*	–
	1	*Salmonella enterica* Serogroup B	–
		Viridans streptococci	
		*Peptostreptococcus* spp.	
	3	*Staphylococcus aureus*	–
	1	*Aeromonas caviae*	–
	1	*Vibrio vulnificus*	–
	2	*Escherichia coli*	–
	2	Viridans streptococci	–
	1	*Streptococcus pneumoniae*	–
	1	*Enterobacter cloacae*	–

*Abbreviations*: *SBP*, spontaneous bacterial peritonitis; *UTI*, urinary tract infection; *ORSA*, oxacillin-resistant *Staphylococcus aureus*.

### Factors associated with risk of 14-day rebleeding

In this study, 76 of the 913 original patients and 12 of the 138 PPS-matched patients experienced rebleeding within 14 days of presentation at the hospital. Table 5 summarizes the RRs of all factors associated with this rebleeding. Among the 913 patients, the factors significantly affecting the RRs of rebleeding were units of blood transfusion (RR: 1.185, 95% CI: 1.055–1.332, *P* = 0.004), albumin levels (RR: 0.500, 95% CI: 0.255–0.981, *P* = 0.004), and treatment with endoscopic variceal ligation (RR: 0.455, 95% CI: 0.240–0.865, *P* = 0.016). Antibiotic prophylaxis did not affect the RR of rebleeding (RR: 0.791, 95% CI: 0.287–2.181, *P* = 0.650) (Table 5 and S3 Table). The risk of 14-day rebleeding did not differ significantly between the patients who received and did not receive antibiotic prophylaxis after PPS adjustment (RR: 0.292, 95% CI: 0.050–1.171, *P* = 0.173). When only the 716 portal hypertensive bleeding patients were included, the risk of 14-day rebleeding did not differ significantly between those who received and did not receive antibiotic prophylaxis (RR: 0.696, 95% CI: 0.223–2.170, *P* = 0.533) as shown in S5 Table.

**Table 5:** Factors associated with risk of rebleeding within 14 days^†^.

Factors		All patients (n = 913)			PPS-matched patients (n = 138)		
		RR	95% CI	*P*	RR	95% CI	*P*
Prophylaxis		0.791	0.287–2.181	0.650	0.292	0.050–1.171	0.173
Age, years		0.992	0.287–2.181	0.483	0.990	0.905–1.083	0.825
Sex, male/female		1.891	0.9807–3.648	0.057	11.969	0.737–194.294	0.081
Prior SBP, y/n		–	–	0.998	–	–	0.999
Ascites, y/n		1.944	0.950–3.980	0.069	0.524	0.022–12.606	0.690
HCCs, y/n		1.131	0.640–1.998	0.672	0.461	0.047–4.564	0.508
Blood transfusion, unit		1.185	1.055–1.332	0.004	1.242	0.861–1.792	0.247
Encephalopathy, y/n		0.690	0.189–2.514	0.573	1.166	0.057–23.708	0.920
Blood pressure, mmHg		0.996	0.987–1.005	0.379	1.104	0.968–1.403	0.320
Hemoglobin, g/L		1.039	0.909–1.187	0.578	1.057	0.649–1.721	0.824
WBC count, ×10^3^/μL		0.927	0.853–1.008	0.076	0.828	0.621–1.015	0.200
Platelet count, ×10^3^/μL		1.002	0.999–1.006	0.240	1.103	0.995–1.032	0.168
Albumin, g/dL		0.500	0.255–0.981	0.004	0.988	0.097–10.106	0.992
ICU admission, y/n		0.914	0.236–3.530	0.896	0.426	0.014–12.662	0.622
MELD score		0.910	0.910–0.831	0.042	0.674	0.447–1.016	0.059
Child Pugh score		0.978	0.657–1.455	0.912	5.330	0.827–34.348	0.078
Etiology of cirrhosis				0.262			0.421
	NBNC	1.000			1.000		
	HBV	1.019	0.411–2.527	0.968	7.168	0.420–122.290	0.174
	HCV	1.830	0.831–4.027	0.133	2.762	0.156–48.989	0.489
	BC	1.750	0.641–4.777	0.275	0.754	0.014–40.663	0.885
Treatment				0.098			0.669
	No treatment	1.000			1.000		
	APC	0.991	0.334–2.944	0.988	–	–	0.999
	EVL	0.455	0.240–0.865	0.016	3.403	0.165–70.014	0.427
	EIS	0.809	0.367–1.782	0.599	6.578	0.311–139.036	0.226
Etiology of bleeding				0.294			0.213
	Portal hypertension	1.000			1.000		
	Peptic ulcer	0.585	0.214–1.599		11.041	0.250–496.156	

^†^ Number of patients with 14-day rebleeding: 76 of all 913 patients and 12 of the 138 PPS-matched patients.

*Abbreviations*: *PPS*, propensity score; *RR*, relative risk; *CI*, confidence interval; *y/n*, yes/no; *SBP*, spontaneous bacterial peritonitis; *HCC*, hepatocellular carcinoma; *WBC*, white blood cell; *ICU*, intensive care unit; *MELD*, model for end-stage liver disease; *NBNC*, negative for both HBV and HCV; *HBV*, hepatitis B virus; *HCV*, hepatitis C virus; *BC*, presence of both HBV and HCV; *APC*, argon plasma coagulation; *EVL*, endoscopic variceal ligation; *EIS*, endoscopic injection sclerosis.

### Factors associated with the risk of 42-day mortality

Among the 913 original patients and 138 PPS-matched patients, 32 and 6 exhibited 42-day mortality, respectively. In the 32 patients, the causes of mortality were potentially multifactorial, with major causes being infection, refractory bleeding, aortic dissection, acute myocardial infarction, and acute on chronic liver failure in 7, 4, 1, 1, and 19 patients, respectively. The 42-day mortality rate was approximately 3.5% in the patients with Child–Pugh A/B cirrhosis and UGI bleeding. As shown in Table 6, except for the presence of HCCs (RR: 5.300, 95% CI: 1.850–15.187, *P* = 0.002) and platelet counts (RR: 1.006, 95% CI: 1.002–1.010, *P* = 0.003), no clinical and laboratory parameters, including antibiotic prophylaxis (RR: 2.710, 95% CI: 0.769–9.524, *P* = 0.121), affected the RR of 42-day mortality. Antibiotic prophylaxis similarly did not significantly affect risk of 42-day mortality in Child–Pugh Class B patient subgroup (RR: 2.681, 95% CI: 0.761–9.434, *P* = 0.125; S3 Table). Because the number of cases of 42-day mortality was low in the PPS-matched cohort, the RRs and their corresponding 95% CIs were incalculable for all factors. However, no factors, including antibiotic prophylaxis, significantly affected the RRs of 42-day mortality after adjustment for PPS (*P* = 0.994–1.000). The use of prophylactic antibiotics did not affect the 42-day mortality (RR: 2.304, 95% CI: 0.558–9.521, *P* = 0.249) when only the 716 portal hypertensive bleeding patients were included for analysis as demonstrated in S6 Table.

**Table 6:** Factors associated with risk of mortality within 42 days^†^.

Factors		All patients (n = 913)			PPS match (n = 138)		
		RR	95% CI	*P*	RR	95% CI	*P*
Prophylaxis, y/n		2.710	0.769–9.524	0.121	–	–	0.998
Age, years		0.991	0.951–1.032	0.656	–	–	0.994
Sex, male/female		0.860	0.295–2.508	0.782	–	–	0.998
Prior SBP, y/n		–	–	0.998	–	–	1.000
Ascites, y/n		0.994	0.329–3.001	0.992	–	–	0.999
HCCs, y/n		5.300	1.850–15.187	0.002	–	–	0.997
Blood transfusion, unit		1.085	0.901–1.305	0.389	–	–	0.999
Encephalopathy, y/n		2.218	0.575–8.557	0.247	–	–	0.999
Blood pressure, mmHg		0.997	0.982–1.013	0.736	–	–	0.997
Hemoglobin, g/L		1.043	0.830–1.309	0.720	–	–	1.000
WBC count, ×10^3^/μL		0.915	0.807–1.038	0.169	–	–	0.999
Platelet count, ×10^3^/μL		1.006	1.002–1.010	0.003	–	–	1.000
Albumin, g/dL		0.407	0.143–1.154	0.091	–	–	1.000
ICU admission, y/n		4.338	0.997–18.867	0.050	–	–	1.000
MELD score		1.098	0.966–1.249	0.153	–	–	0.999
Child Pugh score		1.899	0.972–3.710	0.061	–	–	0.995
Etiology of cirrhosis				0.539			1.000
	NBNC	1.000			1.000		
	HBV	1.189	0.411–2.527	0.824	–	–	0.999
	HCV	0.687	0.153–3.092	0.625	–	–	0.999
	BC	1.849	0.330–10.369	0.485	–	–	1.000
Treatment				0.610			1.000
	No treatment	1.000			1.000		
	APC	0.433	0.073–2.568	0.357	–	–	1.000
	EVL	1.609	0.452–5.731	0.463	–	–	0.999
	EIS	0.908	0.162–5.103	0.913	–	–	0.995
Etiology of bleeding				0.293			1.000
	Portal hypertension	1.000			1.000		
	Peptic ulcer	2.351	0.478–11.500		–	–	

^†^ Number of patients with 42-day mortality: 32 of all 913 patients and 6 of the 138 PPS-matched patients.

*Abbreviations*: *PPS*, propensity score; *RR*, relative risk; *CI*, confidence interval; *y/n*, yes/no; *SBP*, spontaneous bacterial peritonitis; *HCC*, hepatocellular carcinoma; *WBC*, white blood cell; *ICU*, intensive care unit; *MELD*, model for end-stage liver disease; *NBNC*, negative for both HBV and HCV; *HBV*, hepatitis B virus; *HCV*, hepatitis C virus; *BC*, presence of both HBV and HCV; *APC*, argon plasma coagulation; *EVL*, endoscopic variceal ligation; *EIS*, endoscopic injection sclerosis.

## Discussion

Antibiotic overuse is a pressing public health issue in current medical practices because antibiotics can lead to life-threatening complications [8,17]. Thus, an effective antibiotic stewardship program requires restricting antibiotic use to subpopulations at an extremely high bacterial infection risk as well as avoiding inappropriate antibiotic use [17].

In clinical care for patients with cirrhosis, bacterial infection is typically a frequent and severe complication, and prophylactic antibiotic use is an integral part of patient care, particularly in patients with UGI bleeding [18]. Over the preceding three decades, studies investigating the role of antibiotic prophylaxis in patients with cirrhosis and UGI bleeding have demonstrated that antibiotic prophylaxis can reduce rates of bacterial infection, rebleeding, and mortality in patients with cirrhosis and UGI bleeding [6]. Thus, all current practice guidelines and consensus statements recommend antibiotic prophylaxis for all patients with cirrhosis and active UGI bleeding [2–4]. However, most studies on the role of antibiotic prophylaxis in patients with cirrhosis who have UGI bleeding have not considered the differences in bacterial infection risk levels in patients with varying degrees of liver cirrhosis [6].

Patients with Child–Pugh class C cirrhosis have an extremely high risk of bacterial infection, and prophylactic antibiotic use in such patients is justified, because more than half of our class C patients exhibited either documented bacterial infection or clinical signs of bacterial infection within days of UGI bleeding. However, patients with cirrhosis and low liver function impairment, namely those with Child–Pugh class A/B, are at a relatively low risk of bacterial infection, and such infections are generally less severe than those in patients with cirrhosis and high liver function impairment [19]. A recent study in Canada demonstrated that patients with Child–Pugh class A cirrhosis and acute variceal bleeding had low bacterial infection and mortality rates without receiving antibiotic prophylaxis [11]. The prophylactic effects of ceftriaxone were superior to those of cefazolin in terms of preventing infection and rebleeding only in patients categorized as Child–Pugh class B/C; this finding is indirect evidence for the low risk of bacterial infection in patients with Child–Pugh class A cirrhosis [20]. These observations provided the rationale for our study to reappraise the effects of antibiotic prophylaxis in patients with low Child–Pugh scores and UGI bleeding.

In addition to patients with Child–Pugh class C cirrhosis, we excluded those with documented bacterial infection on the day of UGI bleeding, with recent antibiotic use within 1 week before the UGI bleeding episode, and who died within the first 24 h, because these patients may have received or would have inevitably received antibiotic therapy for their clinical conditions. Most of these excluded patients also had Child–Pugh class C cirrhosis.

Most related studies only enrolled patients with variceal bleeding, and most of these patients exhibited alcoholism. By contrast, our study population comprised patients at ratios of 4:1 for viral versus nonviral (majorly alcoholic) cirrhosis and variceal versus peptic ulcer bleeding (Table 1). Our results demonstrated that neither the etiology of cirrhosis nor the cause of UGI bleeding affected the patients’ major outcomes. A recent study suggested that active alcohol drinkers have a high infection risk [12]. By contrast, patients with nonviral cirrhosis had the lower risk for infection in this study. This inconsistency warrants further investigation because our retrospective clinical information did not contain precise data regarding active drinking. Furthermore, the modality of endoscopic therapy did not affect the patient outcomes.

In our study, the proportion of patients who received antibiotic prophylaxis was only 8% (73 of 913 patients with Child–Pugh class A/B cirrhosis)—consistent with a Taiwanese study, where only 6.7% patients with cirrhosis and acute variceal bleeding received prophylactic antibiotics between 2005 and 2006 [21]. Even in Western studies, the percentages of patients receiving prophylactic antibiotics have been <50% [7,10,11]. Thus, antibiotic prophylaxis may not be a common practice in both Western countries and Taiwan in the 2000s, with rates ranging from 6.67% to 49.2% [7,10,12,21].

Our data revealed that 19.6% (165 of 840) of the patients who did not receive antibiotic prophylaxis exhibited clinical signs of infection and received on-demand antibiotics within 14 days of UGI bleeding. Nevertheless, the RR of complications, including bacterial infection, rebleeding, and mortality, was not higher in the patients who did not receive antibiotic prophylaxis than in those who did. Notably, the overall infection rate was 4.9% (45 of 913 patients), which is low, despite the low rate of prophylactic antibiotic use; this suggests that the role of antibiotic prophylaxis is negligible in the patients with Child–Pugh class A/B cirrhosis and UGI bleeding. In the 45 patients with documented bacterial infection, the types and sites of infection were complex and not confined to gram-negative enteric bacteria (Table 4). The heterogeneous nature of the observed bacterial infections implicated that a broad-spectrum antibiotic should be implemented if a prophylactic regimen is intended to cover all potential pathogens.

In our study, patients with peptic ulcer bleeding received a short term intravenous PPI followed by oral PPI for 2–4 months. However, PPI use was suggested to increase the risk of SBP [22]. Although one multicenter prospective study indicated that PPI therapy does not increase the incidence of SBP in cirrhosis, PPIs should be administered after cautious evaluation of the indications in cirrhotic patients with UGI bleeding [23].

This was a retrospective study and thus has several limitations: First, we could not control for the confounding parameters; to resolve this, PPS matching was performed. Second, the prescriptions of antibiotics provided to our patients included cefazolin, cefazolin plus gentamicin, and cefuroxime and ceftriaxone; therefore, the use of antibiotic prophylaxis did not completely fit with the actual guidelines, which recommend quinolones or third-generation cephalosporins for 5–7 days after UGI bleeding. Third, no routine screening was performed for bacterial infections and therefore some occult infections may have been overlooked. Nevertheless, our findings revealed that even without routine infection workup and antibiotic use, the rate of clinically relevant infection was low. Fourth, distinguishing patients with Child–Pugh class B cirrhosis from those with class C cirrhosis can be challenging in a retrospective analysis because the factors, such as hepatic encephalopathy and ascites, are fairly subjective. Fifth, since transjugular intrahepatic portosystemic shunt has been unavailable in the study site, the impact of this therapeutic approach on the outcomes was lacking. Finally, data regarding the effects of prior bleeding episodes on patient outcomes could not be obtained because we enrolled patients with only initial UGI bleeding episodes.

Our study, nevertheless, suggests a low risk of bacterial infection in patients with low Child–Pugh scores and UGI bleeding, thus providing a rationale for additional randomized control trials comparing the differences in the effects of prophylactic versus on-demand antibiotic use in patients with low Child–Pugh scores and UGI bleeding.

## Conclusions

In patients with Child–Pugh class A/B cirrhosis and UGI bleeding, bacterial infection risk is low, and antibiotic prophylaxis has minor effects on the major outcomes. To develop an effective antibiotic stewardship program and minimize side effects, on-demand antibiotic use may be an optimal strategy for these patients. Additional randomized control trials to confirm this finding are warranted.

## Supporting information

S1 Table Clinical outcomes of Child–Pugh Class A and B cirrhosis patients, with and without prophylactic antibiotic use. (DOCX)

S2 Table Baseline patient characteristics in portal hypertensive patient subgroup. (DOCX)

S3 Table Relative risks of clinical outcomes with respect to prophylactic antibiotic use in Child–Pugh Class A and B cirrhosis patients. (DOCX)

S4 Table Factors associated with bacterial infection risk within 14 days in portal hypertensive patient subgroup. (DOCX)

S5 Table Factors associated with risk of rebleeding within 14 days in portal hypertensive patient subgroup. (DOCX)

S6 Table Factors associated with risk of mortality within 42 days in portal hypertensive patient subgroup. (DOCX)

S7 Table Patient data of this study cohort. (XLSX)
