# Supplementary material for: Limited effects of antibiotic prophylaxis in patients with Child–Pugh class A/B cirrhosis and upper gastrointestinal bleeding
Source: PLoS One. 2020 Feb 21;15(2):e0229101. doi: 10.1371/journal.pone.0229101 (PMC7034903; doi:10.1371/journal.pone.0229101)
Supplement: S1 Table — (DOCX) [file pone.0229101.s001.docx]

**Supporting Information**

**Supplementary Table 1.** Clinical outcomes of Child–Pugh Class A and B cirrhosis patients, with and without prophylactic antibiotic use.

|  | **Child–Pugh Class A** | | | **Child–Pugh Class B** | | |
| --- | --- | --- | --- | --- | --- | --- |
|  | **Prophylaxis**  **(n=18)** | **No Prophylaxis**  **(n=240)** | ***P***^†^ | **Prophylaxis**  **(n=55)** | **No Prophylaxis**  **(n=600)** | ***P*** |
| Infection within 14 days, *n* (%) | 0 (0%) | 7 (2.9%) | 1.000 | 5 (9.1%) | 33 (5.5%) | 0.238 |
| Rebleeding within 14 days, *n* (%) | 0 (0%) | 16 (6.7%) | 0.612 | 5 (9.1%) | 55 (9.2%) | 0.985 |
| Mortality within 42 days, *n* (%) | 0 (0%) | 0 (0%) | – | 5 (9.1%) | 27 (4.5%) | 0.177 |

^†^ Comparison between antibiotic prophylaxis and no prophylaxis groups in Child–Pugh Class A and Class B patients, respectively.
