# Supplementary material for: Limited effects of antibiotic prophylaxis in patients with Child–Pugh class A/B cirrhosis and upper gastrointestinal bleeding
Source: PLoS One. 2020 Feb 21;15(2):e0229101. doi: 10.1371/journal.pone.0229101 (PMC7034903; doi:10.1371/journal.pone.0229101)
Supplement: S2 Table — (DOCX) [file pone.0229101.s002.docx]

**Supporting Information**

**Supplementary Table 2.** Baseline patient characteristics in portal hypertensive patient subgroup.

| **Patient characteristics** | **Prophylaxis**  **(*n* = 68)** | **No prophylaxis**  **(*n* = 648)** | **All PTH patients**  **(*n* = 716)** | ***P*** ^†^ |
| --- | --- | --- | --- | --- |
| Age, mean ± SD (years) | 55.41 ± 12.41 | 59.04 ± 13.15 | 58.69 ± 13.11 | 0.030 |
| Sex, male *n* (%) | 52 (76.5) | 449 (69.3) | 501 (70.0) | 0.219 |
| HCC, *n* (%) | 17 (25.0) | 227 (35.0) | 244 (34.1) | 0.097 |
| Blood transfused in 48 h, unit | 3.43 ± 2.70 | 2.54 ± 2.49 | 2.63 ± 2.52 | 0.006 |
| Ascites, *n* (%) | 31 (45.6) | 279 (43.1) | 310 (43.3) | 0.688 |
| Hepatic encephalopathy, *n* (%) | 4 (5.9) | 31 (4.8) | 35 (4.9) | 0.565 |
| Prior SBP, *n* (%) | 0 (0.0) | 12 (1.9) | 12 (1.7) | 0.617 |
| Etiology of cirrhosis, *n* (%) |  |  |  | 0.298 |
| HBV | 20 (29.4) | 136 (21.0) | 156 (21.8) |  |
| HCV | 27 (39.7) | 318 (49.1) | 345 (48.2) |  |
| BC | 8 (11.8) | 59 (9.1) | 67 (9.4) |  |
| NBNC | 13 (19.1) | 135 (20.8) | 148 (20.7) |  |
| Platelet count, ×10^3^/µL | 116.71 ± 70.18 | 111.07 ± 72.60 | 111.60 ± 72.35 | 0.541 |
| White blood cell count, ×10^3^/µL | 10.00 ± 4.36 | 8.01 ± 4.05 | 8.20 ± 4.12 | <0.001 |
| Hemoglobin, g/L | 9.35 ± 2.27 | 9.23 ± 2.38 | 9.24 ± 2.37 | 0.687 |
| International normalized ratio | 1.31 ± 0.17 | 1.29 ± 0.51 | 1.29 ± 0.49 | 0.362 |
| Sodium, mEq/L | 136.75 ± 3.77 | 136.53 ± 10.85 | 136.55 ± 10.38 | 0.866 |
| Creatinine, mg/L | 1.08 ± 0.40 | 1.16 ± 0.88 | 1.15 ± 0.85 | 0.473 |
| Bilirubin, mg/dL | 1.79 ± 1.06 | 1.79 ± 1.18 | 1.79 ± 1.17 | 0.997 |
| ALT, IU/L | 46.51 ± 24.84 | 66.68 ± 138.23 | 64.77 ± 131.86 | 0.234 |
| Albumin, g/dL | 2.98 ± 0.47 | 2.91 ± 0.54 | 2.92 ± 0.54 | 0.306 |
| Systolic blood pressure, mmHg | 113.21 ± 27.25 | 120.20 ± 31.27 | 119.50 ± 30.94 | 0.077 |
| Heart rate, beats/min | 104.71 ± 21.74 | 101.14 ± 21.60 | 101.49 ± 21.63 | 0.197 |
| Hospitalization days | 6.85 ± 4.06 | 6.59 ± 5.07 | 6.62 ± 4.98 | 0.682 |
| ICU admission, *n* (%) | 5 (7.4) | 15 (2.3) | 26 (2.84) | <0.001 |
| MELD score | 12.59 ± 3.49 | 12.33 ± 3.55 | 12.35 ± 3.55 | 0.566 |
| Child–Pugh score | 7.31 ± 1.20 | 7.33 ± 1.20 | 7.33 ± 1.20 | 0.896 |
| Child–Pugh class A/B, *n* (%) | 17/51  (25.0/75.0) | 179/469  (27.6/72.4) | 196/520  (27.4/72.6) | 0.644 |
| Infection within 14 days, *n* (%) | 3 (4.4) | 33 (5.1) | 36 (5.0) | 1.000 |
| Rebleeding within 14 days, *n* (%) | 4 (5.9) | 58 (9.0) | 62 (8.7) | 0.392 |
| Mortality within 42 days, *n* (%) | 4 (5.9) | 22 (3.4) | 26 (3.6) | 0.401 |

^†^ Comparison between antibiotic prophylaxis and no prophylaxis groups

*Abbreviations: PTH*, portal hypertension; *SD*, standard deviation; *HCC*, hepatocellular carcinoma; *SBP*, spontaneous bacterial peritonitis; *HBV*, hepatitis B virus; *HCV*, hepatitis C virus; *BC*, presence of both HBV and HCV; *NBNC*, negative for both HBV and HCV; *ALT*, alanine aminotransferase; *ICU*, intensive care unit; *MELD*, model for end-stage liver disease.
