# Supplementary material for: Limited effects of antibiotic prophylaxis in patients with Child–Pugh class A/B cirrhosis and upper gastrointestinal bleeding
Source: PLoS One. 2020 Feb 21;15(2):e0229101. doi: 10.1371/journal.pone.0229101 (PMC7034903; doi:10.1371/journal.pone.0229101)
Supplement: S3 Table — (DOCX) [file pone.0229101.s003.docx]

**Supporting Information**

**Supplementary Table 3.** Relative risks of clinical outcomes with respect to prophylactic antibiotic use in Child–Pugh Class A and B cirrhosis patients.^†^

|  | **Child–Pugh Class A (n=258)** | | | **Child–Pugh Class B (n=655)** | | |
| --- | --- | --- | --- | --- | --- | --- |
|  | **RR** | **CI** | ***P*** | **RR** | **CI** | ***P*** |
| Risk of infection within 14 days | – | – | 0.999 | 1.122 | 0.345–3.650 | 0.848 |
| Risk of rebleeding within 14 days | – | – | 0.998 | 1.037 | 0.362–2.976 | 0.946 |
| Risk of mortality within 42 days | – | – | – | 2.681 | 0.761–9.434 | 0.125 |

^†^A multivariate logistic regression model was used to identify independent risk factors of each clinical outcome. Only the relative risks with respect to prophylactic antibiotic use are presented in this table.

*Abbreviations: RR*, relative risk; *CI*, confidence interval.
