# Supplementary material for: Limited effects of antibiotic prophylaxis in patients with Child–Pugh class A/B cirrhosis and upper gastrointestinal bleeding
Source: PLoS One. 2020 Feb 21;15(2):e0229101. doi: 10.1371/journal.pone.0229101 (PMC7034903; doi:10.1371/journal.pone.0229101)
Supplement: S4 Table — (DOCX) [file pone.0229101.s004.docx]

**Supporting Information**

**Supplementary Table 4.** Factors associated with bacterial infection risk within 14 days in portal hypertensive patient subgroup.^†^

| **Factors** | **PTH patients (*n* = 716)** | | | **PPS-matched patients (*n* = 134)** | | |
| --- | --- | --- | --- | --- | --- | --- |
|  | RR | 95% CI | *P* | RR | 95% CI | *P* |
| Prophylaxis, y/n | 0.827 | 0.233–2.937 | 0.769 | 0.242 | 0.046–1.285 | 0.096 |
| Age, years | 1.019 | 0.985–1.053 | 0.281 | 1.044 | 0.975–1.118 | 0.218 |
| Sex, male/female | 0.782 | 0.336–1.818 | 0.568 | 2.487 | 0.295–20.947 | 0.402 |
| Prior SBP, y/n | – | – | 0.999 | – | – | 0.999 |
| Ascites, y/n | 1.490 | 0.575–3.859 | 0.412 | 1.102 | 0.138–8.818 | 0.927 |
| HCCs, y/n | 0.938 | 0.427–2.061 | 0.874 | 1.451 | 0.181–11.636 | 0.726 |
| Blood transfusion, unit | 1.061 | 0.903–1.247 | 0.470 | 1.058 | 0.763–1.467 | 0.737 |
| Encephalopathy, y/n | 1.203 | 0.241–6.014 | 0.822 | 3.047 | 0.193–48.067 | 0.428 |
| Blood pressure, mmHg | 1.002 | 0.991–1.014 | 0.693 | 0.989 | 0.959–1.019 | 0.464 |
| Hemoglobin, g/L | 1.127 | 0.922–1.377 | 0.244 | 1.204 | 0.755–1.919 | 0.435 |
| WBC count, ×10^3^/µL | 1.008 | 0.908–1.119 | 0.879 | 0.896 | 0.716–1.121 | 0.336 |
| Platelet count, ×10^3^/µL | 0.992 | 0.983–1.001 | 0.073 | 0.987 | 0.964–1.010 | 0.274 |
| Albumin, g/dL | 0.355 | 0.142–0.890 | 0.027 | 0.155 | 0.019–1.262 | 0.081 |
| ICU admission, y/n | 0.707 | 0.114–4.368 | 0.709 | 0.399 | 0.010–16.346 | 0.628 |
| MELD score | 1.150 | 1.038–1.275 | 0.008 | 1.005 | 0.743–1.360 | 0.974 |
| Child Pugh score | 0.794 | 0.472–1.337 | 0.385 | 0.766 | 0.240–2.443 | 0.652 |
| Etiology of cirrhosis |  |  | 0.716 |  |  | 0.953 |
| NBNC | 1.000 |  |  | 1.000 |  |  |
| HBV | 1.988 | 0.397–9.950 | 0.493 | – | – | 0.999 |
| HCV | 1.636 | 0.349–7.673 | 0.640 | – | – | 0.999 |
| BC | 1.133 | 0.189–6.784 | 0.892 | – | – | 0.999 |
| Treatment |  |  | 0.415 |  |  | 0.939 |
| No treatment | 1.000 |  |  | 1.000 |  |  |
| APC | 2.451 | 0.396–15.158 | 0.772 | – | – | 0.999 |
| EVL | 0.999 | 0.413–2.420 | 0.164 | 0.837 | 0.157–4.471 | 0.568 |
| EIS | 1.872 | 0.639–5.487 | 0.253 | 0.514 | 0.045–5.812 | 0.590 |

^†^ Number of patients with 14-day infection: 36 of all 716 patients and 12 of the 134 PPS-matched patients.

*Abbreviations: PTH*, portal hypertension; *PPS*, propensity score; *RR*, relative risk; *CI*, confidence interval; *y/n*, yes/no; *SBP*, spontaneous bacterial peritonitis; *HCC*, hepatocellular carcinoma; *WBC*, white blood cell; *ICU*, intensive care unit; *MELD*, model for end-stage liver disease; *NBNC*, negative for both HBV and HCV; *HBV*, hepatitis B virus; *HCV*, hepatitis C virus; *BC*, presence of both HBV and HCV; *APC*, argon plasma coagulation; *EVL*, endoscopic variceal ligation; *EIS*, endoscopic injection sclerosis.
