# Supplementary material for: Limited effects of antibiotic prophylaxis in patients with Child–Pugh class A/B cirrhosis and upper gastrointestinal bleeding
Source: PLoS One. 2020 Feb 21;15(2):e0229101. doi: 10.1371/journal.pone.0229101 (PMC7034903; doi:10.1371/journal.pone.0229101)
Supplement: S5 Table — (DOCX) [file pone.0229101.s005.docx]

**Supporting Information**

**Supplementary Table 5.** Factors associated with risk of rebleeding within 14 days in portal hypertensive patient subgroup.^†^

| **Factors** | **PTH patients (n = 716)** | | | **PPS-matched patients (n = 134)** | | |
| --- | --- | --- | --- | --- | --- | --- |
|  | RR | 95% CI | *P* | RR | 95% CI | *P* |
| Prophylaxis | 0.696 | 0.223–2.170 | 0.533 | 0.283 | 0.022–3.617 | 0.331 |
| Age, years | 0.985 | 0.959–1.012 | 0.276 | 1.024 | 0.911–1.150 | 0.695 |
| Sex, male/female | 2.306 | 1.084–4.906 | 0.030 | 3.923 | 0.241–63.818 | 0.337 |
| Prior SBP, y/n | – | – | 0.999 | – | – | 0.999 |
| Ascites, y/n | 2.115 | 0.924–4.842 | 0.076 | 0.665 | 0.012–36.515 | 0.842 |
| HCCs, y/n | 1.494 | 0.640–1.998 | 0.672 | 13.857 | 0.465–412.852 | 0.129 |
| Blood transfusion, unit | 1.173 | 1.031–1.334 | 0.015 | 1.806 | 0.729–4.475 | 0.202 |
| Encephalopathy, y/n | 0.863 | 0.225–3.307 | 0.829 | 6.458 | 0.227–183.420 | 0.275 |
| Blood pressure, mmHg | 0.993 | 0.983–1.004 | 0.201 | 0.960 | 0.911–1.102 | 0.126 |
| Hemoglobin, g/L | 1.029 | 0.887–1.194 | 0.705 | 2.127 | 0.774–5.848 | 0.143 |
| WBC count, ×10^3^/µL | 0.892 | 0.806–0.987 | 0.027 | 0.604 | 0.326–1.116 | 0.108 |
| Platelet count, ×10^3^/µL | 1.002 | 0.998–1.007 | 0.364 | 0.995 | 0.975–1.015 | 0.624 |
| Albumin, g/dL | 0.510 | 0.236–1.105 | 0.088 | 0.794 | 0.016–40.023 | 0.908 |
| ICU admission, y/n | 1.617 | 0.337–7.751 | 0.548 | 2.041 | 0.006–695.852 | 0.810 |
| MELD score | 0.876 | 0.783–0.981 | 0.022 | 0.659 | 0.397–1.094 | 0.107 |
| Child Pugh score | 1.086 | 0.682–1.730 | 0.727 | 2.424 | 0.354–16.586 | 0.367 |
| Etiology of cirrhosis |  |  | 0.109 |  |  | 0.837 |
| NBNC | 1.000 |  |  | 1.000 |  |  |
| HBV | 1.105 | 0.388–3.148 | 0.245 | – | – | 0.753 |
| HCV | 2.502 | 0.984–6.362 | 0.679 | – | – | 0.617 |
| BC | 2.055 | 0.632–6.684 | 0.231 | – | – | 0.997 |
| Treatment |  |  | 0.072 |  |  | 0.851 |
| No treatment | 1.000 |  |  | 1.000 |  |  |
| APC | 0.767 | 0.082–7.200 | 0.956 | – | – | 0.999 |
| EVL | 0.413 | 0.188–0.911 | 0.168 | 0.420 | 0.031–5.710 | 0.474 |
| EIS | 0.720 | 0.313–1.658 | 0.440 | 0.162 | 0.003–10.018 | 0.387 |

^†^ Number of patients with 14-day rebleeding: 62 of all 716 patients and 9 of the 138 PPS-matched patients.

*Abbreviations: PTH*, portal hypertension; *PPS*, propensity score; *RR*, relative risk; *CI*, confidence interval; *y/n*, yes/no; *SBP*, spontaneous bacterial peritonitis; *HCC*, hepatocellular carcinoma; *WBC*, white blood cell; *ICU*, intensive care unit; *MELD*, model for end-stage liver disease; *NBNC*, negative for both HBV and HCV; *HBV*, hepatitis B virus; *HCV*, hepatitis C virus; *BC*, presence of both HBV and HCV; *APC*, argon plasma coagulation; *EVL*, endoscopic variceal ligation; *EIS*, endoscopic injection sclerosis.
