# Supplementary material for: Limited effects of antibiotic prophylaxis in patients with Child–Pugh class A/B cirrhosis and upper gastrointestinal bleeding
Source: PLoS One. 2020 Feb 21;15(2):e0229101. doi: 10.1371/journal.pone.0229101 (PMC7034903; doi:10.1371/journal.pone.0229101)
Supplement: S6 Table — (DOCX) [file pone.0229101.s006.docx]

**Supporting Information**

**Supplementary Table 6.** Factors associated with risk of mortality within 42 days in portal hypertensive patient subgroup.^†^

| **Factors** | **All patients (n = 716)** | | | **PPS match (n = 134)** | | |
| --- | --- | --- | --- | --- | --- | --- |
|  | RR | 95% CI | *P* | RR | 95% CI | *P* |
| Prophylaxis, y/n | 2.304 | 0.558–9.521 | 0.249 | – | – | 0.996 |
| Age, years | 0.980 | 0.934–1.029 | 0.427 | – | – | 0.998 |
| Sex, male/female | 0.830 | 0.237–2.910 | 0.771 | – | – | 0.997 |
| Prior SBP, y/n | – | – | 0.999 | – | – | 0.999 |
| Ascites, y/n | 0.678 | 0.172–2.670 | 0.579 | – | – | 1.000 |
| HCCs, y/n | 8.343 | 2.206–31.555 | 0.002 | – | – | 0.997 |
| Blood transfusion, unit | 1.063 | 0.862–1.310 | 0.569 | – | – | 0.998 |
| Encephalopathy, y/n | 3.220 | 0.703–14.749 | 0.132 | – | – | 1.000 |
| Blood pressure, mmHg | 0.990 | 0.971–1.009 | 0.288 | – | – | 0.999 |
| Hemoglobin, g/L | 0.881 | 0.674–1.153 | 0.356 | – | – | 0.998 |
| WBC count, ×10^3^/µL | 0.957 | 0.826–1.110 | 0.562 | – | – | 0.998 |
| Platelet count, ×10^3^/µL | 1.005 | 1.001–1.010 | 0.014 | – | – | 0.998 |
| Albumin, g/dL | 0.453 | 0.138–1.485 | 0.191 | – | – | 0.996 |
| ICU admission, y/n | 0.223 | 0.037–1.336 | 0.101 | – | – | 0.999 |
| MELD score | 1.119 | 0.964–1.299 | 0.140 | – | – | 0.999 |
| Child Pugh score | 2.090 | 0.925–4.724 | 0.076 | – | – | 1.000 |
| Etiology of cirrhosis |  |  | 0.431 |  |  | 0.999 |
| NBNC | 1.000 |  |  | 1.000 |  |  |
| HBV | 3.279 | 0.571–18.817 | 0.408 | – | – | 0.998 |
| HCV | 1.295 | 0.208–8.074 | 0.838 | – | – | 0.999 |
| BC | 1.567 | 0.140–17.614 | 0.485 | – | – | 0.998 |
| Treatment |  |  | 0.871 |  |  | 1.000 |
| No treatment | 1.000 |  |  | 1.000 |  |  |
| APC | – | – | 0.999 | – | – | 0.999 |
| EVL | 1.717 | 0.388–7.598 | 0.643 | – | – | 1.000 |
| EIS | 1.208 | 0.179–8.156 | 0.846 | – | – | 0.998 |

^†^ Number of patients with 42-day mortality: 26 of all 716 patients and 4 of the 134 PPS-matched patients.

*Abbreviations: PTH*, portal hypertension; *PPS*, propensity score; *RR*, relative risk; *CI*, confidence interval; *y/n*, yes/no; *SBP*, spontaneous bacterial peritonitis; *HCC*, hepatocellular carcinoma; *WBC*, white blood cell; *ICU*, intensive care unit; *MELD*, model for end-stage liver disease; *NBNC*, negative for both HBV and HCV; *HBV*, hepatitis B virus; *HCV*, hepatitis C virus; *BC*, presence of both HBV and HCV; *APC*, argon plasma coagulation; *EVL*, endoscopic variceal ligation; *EIS*, endoscopic injection sclerosis.
